# Supplementary material for: Early life infection and proinflammatory, atherogenic metabolomic and lipidomic profiles in infancy: a population-based cohort study
Source: eLife. 2022 May 10;11:e75170. doi: 10.7554/eLife.75170 (PMC9090335; doi:10.7554/eLife.75170)
Supplement: Supplementary file 3. — (A) Difference in log number of parent-reported infections from birth to 6 months of age for each SD increase in log serum NMR metabolomic measure at birth. (B) Difference in log number of parent-reported infections from 6 to 12 months of age for each SD increase in 6-month log plasma NMR metabolomic measure. (C) Difference in log number of parent-reported infections from birth to 6 months of age for each SD increase in log serum LC/MS lipidomic measure at birth. (D) Difference in log number of parent-reported infections from 6 to 12 months of age for each SD increase in 6-month log serum LC/MS lipidomic measure. [file elife-75170-supp3.pdf]

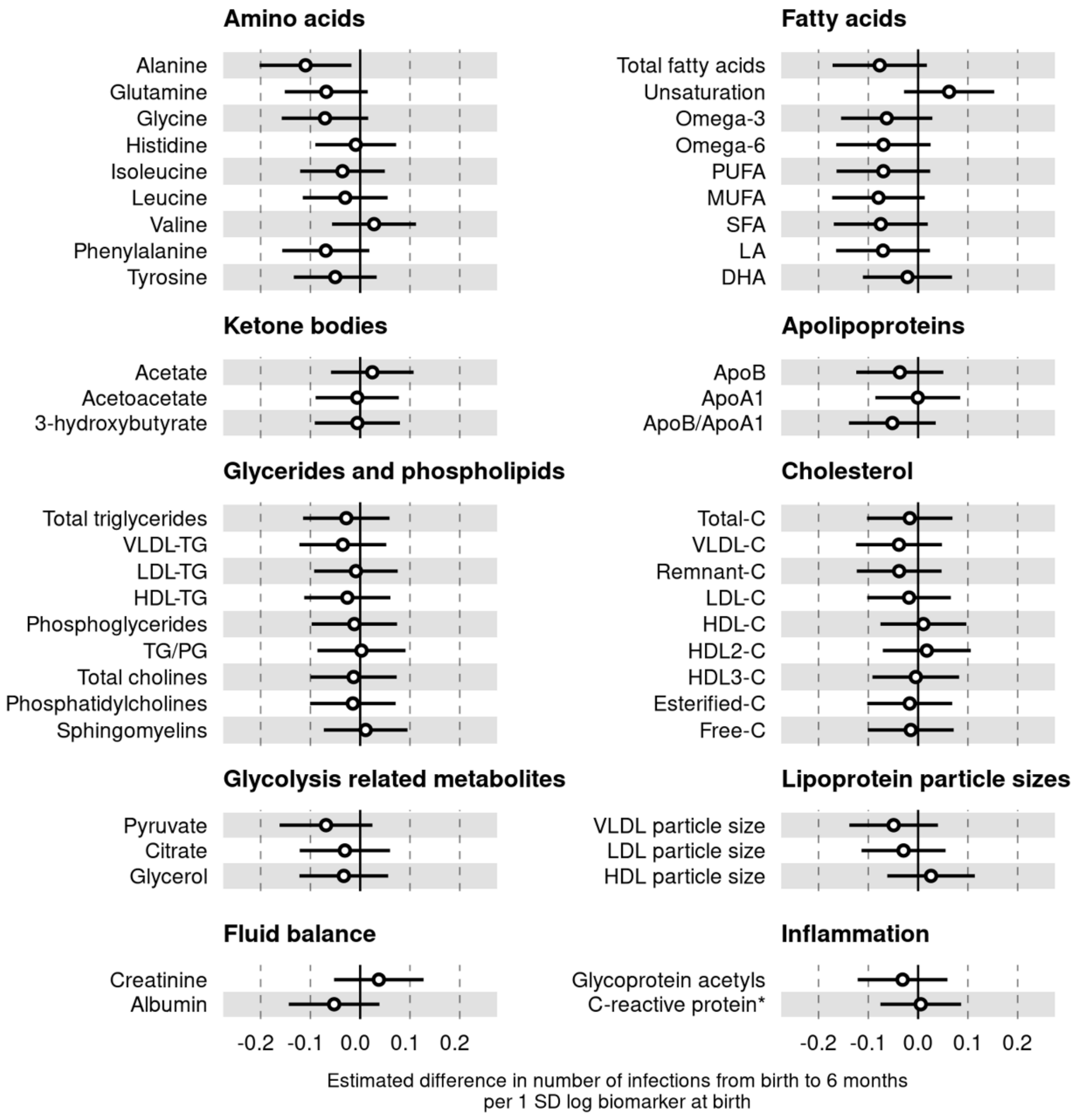

Supplementary File 3A. Difference in log number of parent-reported infections from birth to 6 months of age for each SD increase in log serum NMR metabolomic measure at birth. Error bars are 95% confidence intervals. Closed points represent adjusted p-value<0.05. Models were adjusted for infant age, sex, gestational age, birth weight, mode of delivery, maternal household income, smoking during pregnancy, and breastfeeding status.

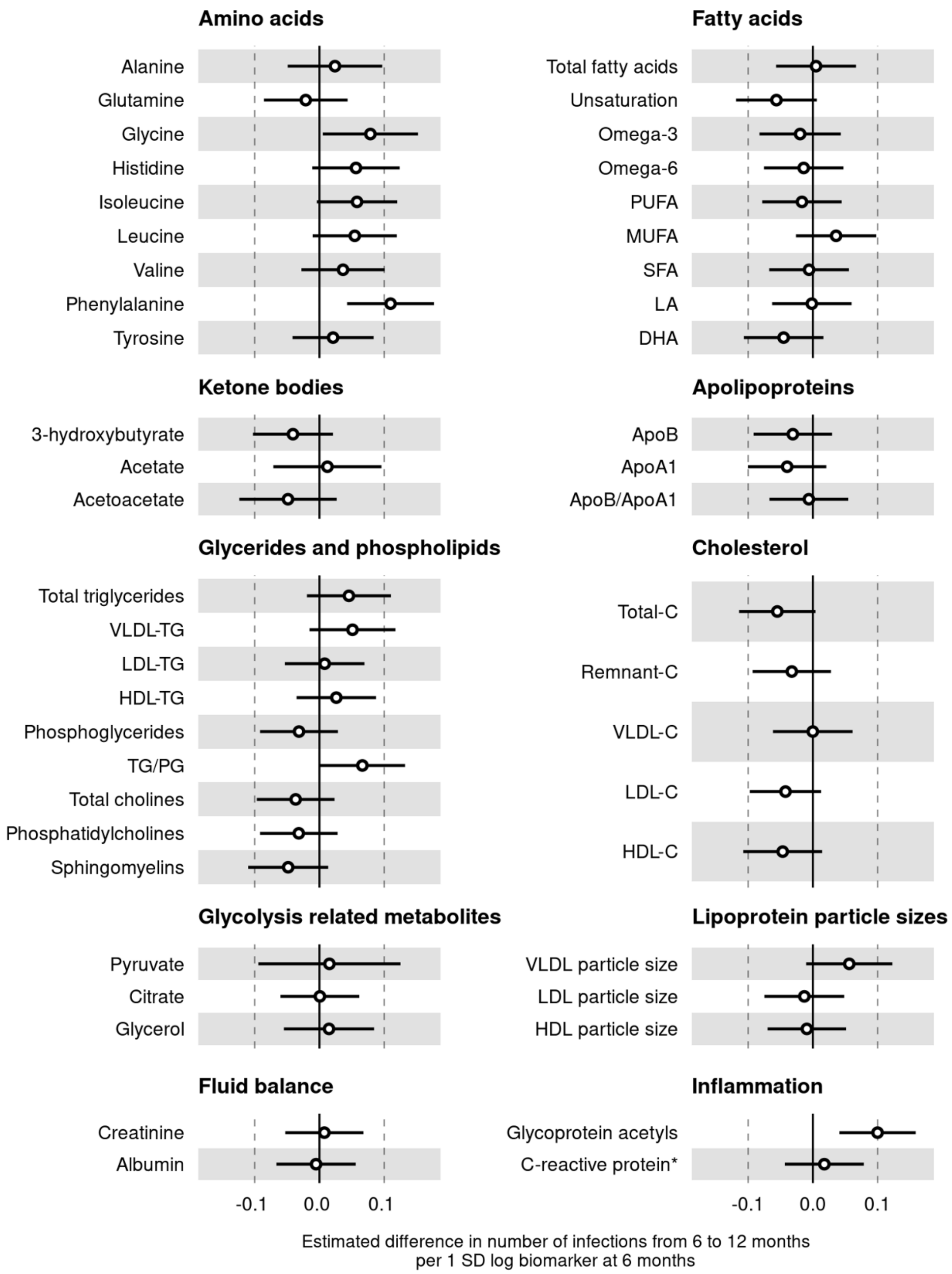

Supplementary File 3B. Difference in log number of parent-reported infections from 6 to 12 months of age for each SD increase in 6-month log plasma NMR metabolomic measure. Error bars are 95% confidence intervals. Closed points represent adjusted p-value<0.05. Models were adjusted for number of infections from birth to 6 months of age, infant age, sex, gestational age, birth weight, mode of delivery, maternal household income, smoking during pregnancy, breastfeeding status, and sample processing time.

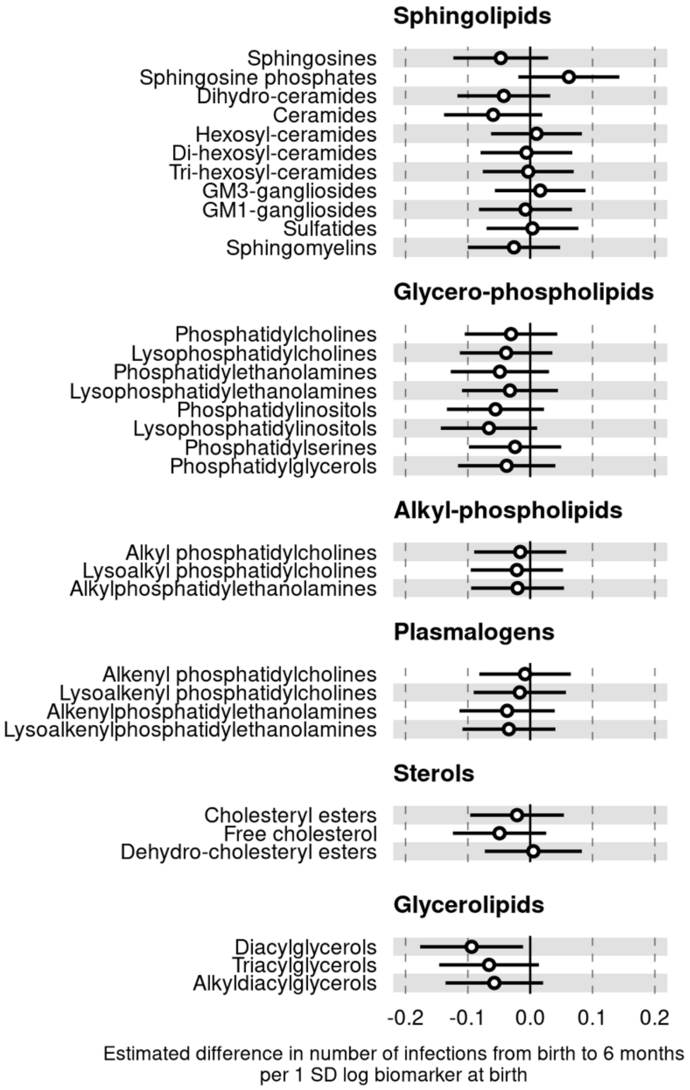

Supplementary File 3C. Difference in log number of parent-reported infections from birth to 6 months of age for each SD increase in log serum LC/MS lipidomic measure at birth. Error bars are 95% confidence intervals. Closed points represent adjusted p-value<0.05. Models were adjusted for infant age, sex, gestational age, birth weight, mode of delivery, maternal household income, smoking during pregnancy, and breastfeeding status.

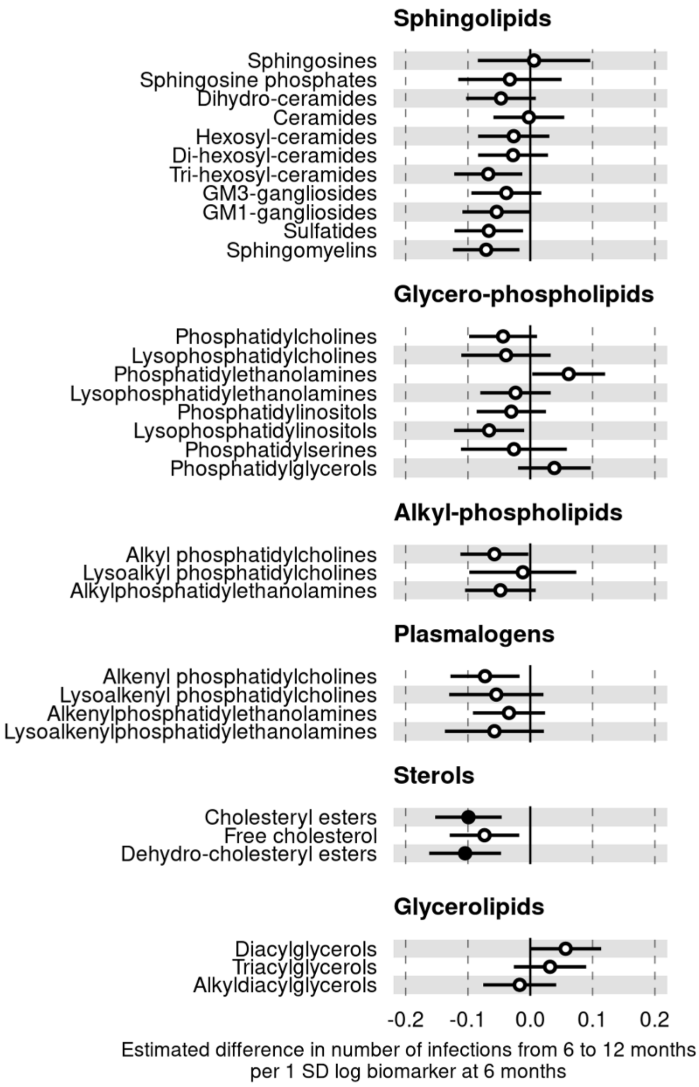

Supplementary File 3D. Difference in log number of parent-reported infections from 6 to 12 months of age for each SD increase in 6-month log serum LC/MS lipidomic measure. Error bars are 95% confidence intervals. Closed points represent adjusted p-value<0.05. Models were adjusted for number of infections from birth to 6 months of age, infant age, sex, gestational age, birth weight, mode of delivery, maternal household income, smoking during pregnancy, breastfeeding status, and sample processing time.
